# Crumpling-based soft metamaterials: the effects of sheet pore size and porosity

**DOI:** 10.1038/s41598-017-12821-6

**Published:** 2017-10-12

**Authors:** M. J. Mirzaali, M. Habibi, S. Janbaz, L. Vergani, A. A. Zadpoor

**Affiliations:** 1 Department of Mechanical Engineering, Politecnico di Milano, Via La Masa 1, 20156 Milano, Italy; 2 Department of Biomechanical Engineering, Faculty of Mechanical, Maritime, and Materials Engineering, Delft University of Technology (TU Delft), Mekelweg 2, 2628 CD Delft, The Netherlands; 3 Physics and Physical Chemistry of Foods, Department of Agrotechnology and Food Sciences, Wageningen University, Wageningen, The Netherlands

## Abstract

Crumpled-based materials are relatively easy to fabricate and show robust mechanical properties for practical applications, including meta-biomaterials design aimed for improved tissue regeneration. For such requests, however, the structure needs to be porous. We introduce a crumpled holey thin sheet as a robust bio-metamaterial and measure the mechanical response of a crumpled holey thin Mylar sheet as a function of the hole size and hole area fraction. We also study the formation of patterns of crease lines and ridges. The area fraction largely dominated the crumpling mechanism. We also show, the crumpling exponents slightly increases with increasing the hole area fraction and the total perimeter of the holes. Finally, hole edges were found to limit and guide the propagation of crease lines and ridges.

## Introduction

Crumpling and folding of thin-wall objects are frequently encountered in nature, technology, and everyday life. Examples are morphogenesis of brain cortex^1^, flower buds^2^, and crumpled graphene^3^. The physics of crumpling has recently received increasing attention^4^. In particular, several studies have tried to reveal the relationship between the topological features induced during the crumpling process and the level of compaction and the associated forces^4–9^. The high strength and energy absorbance capabilities of crumpled structures have been studied as well^8,10^.

Crumpling could repeat itself under confined and controlled conditions. Understanding the physics of crumpling in such controlled experiments could be helpful for designing crumpling-based metamaterials, where crumpled structures act as the building blocks. Crumpling a thin sheet create a low weight structure with significant stiffness and robust mechanical response against disorder and noise which are abundant in real world applications^4^. Inherently random geometric configurations in crumpled-based metamaterials make them less sensitive to topological and geometrical imperfections as compared to ordered metamaterials whose material properties are strongly dependent on precisely controlled topological designs. Moreover, controlled crumpling provides a way for transforming the shape of initially flat structures to a complex three-dimensional topology that gives rise to the desired metamaterial properties. The initially flat state of the matter could then be used to decorate the surface of the sheet with functionality-inducing features^11^. Therefore, crumpled structures are promising candidates for designing bio-metamaterial. However, their morphology and mechanical response need to be studied in order to tailor the desired properties. In the context of meta-biomaterials, i.e. metamaterials used for improving tissue regeneration, the surface features could, for example, be nano-patterned that guide stem cell differentiation and fate^12,13^. For such applications, however, the crumpled shape of the meta-biomaterial should be porous to allow for cell migration, oxygenation, and nutrition. Starting from a holey thin sheet would allow for obtaining a porous crumpled shape with pore sizes and porosities dependent on the degree of crumpling and the area fraction of the sheet. This could provide a single-step approach for fabricating porous meta-biomaterials with (nano-) patterned surfaces.

In the recent work, the effect of material properties on morphology and mechanical response of a crumpled thin sheet was studied in detail experimentally and has been shown that there is a strong link between the morphology and mechanical response of the crumpled structure^4^. However, introducing holes to a flat sheet before crumpling can considerably change the crumpling mechanism and make it more complex. As far as we know, crumpling behavior of holey thin sheets has never been studied before, and the effects of hole size and hole area fraction on the physics of crumpling are unknown. In this paper, for the first time, we investigate the effects of hole size and area fraction on the crumpling behavior of thin elastic sheets in order to design bio-metamaterials.

## Experimental Procedure

**Figure 1:**
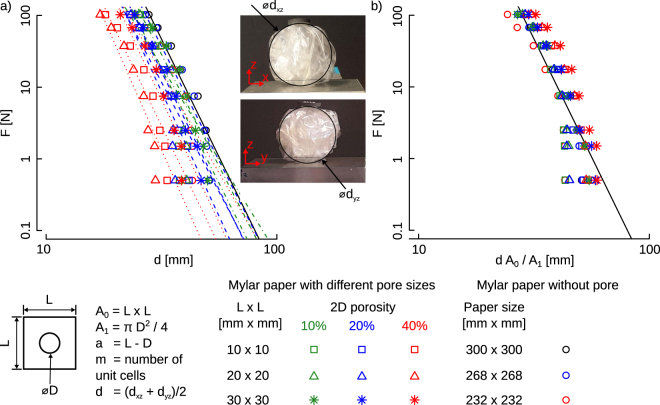
Comparison of force, *F*, with respect to the deformed dimension, *d*, (**a**) and normalized deformation (**b**) for porous and non-porous Mylar sheets. Data are plotted on the logarithmic scale. Specific geometrical parameters for each unit cell are determined in Table 1. Points represent mean value of the compaction level, *d*, in these plots.

We have used Mylar sheets (CLAYRTON’S^®^) of 30 microns. Samples were divided into four different categories: i) Mylar sheets without any hole, in three different sizes: 300 mm × 300 mm, 268 mm × 268 mm, and 232 mm × 232 mm. The 300 mm by 300 mm samples were regarded as the reference samples while in the other two sizes in this category we have reduction of 20% and 40% of the area respect to the reference sample. ii) Mylar sheets with a hole area fraction of 10%, meaning that the total sheet area was ten times larger than the area of the holes. For the samples in this group, three unit cells size, *L*, of 30 mm × 30 mm, 20 mm × 20 mm and 10 mm × 10 mm with different hole sizes were chosen. iii) Mylar sheets with an initial hole area fraction of 20%. Three different unit cell sizes, i.e. 10 mm by 10 mm, 20 mm by 20 mm, and 30 mm by 30 mm, with various hole sizes were considered. iv) Mylar sheets with an initial hole area fraction of 40%. Similar to the samples in groups ii and iii, three hole sizes have been taken into account in this group. The total size of the sheet was kept constant (i.e. 300 mm × 300 mm) for the samples in groups ii to iv. The pores, *D*, (see Fig. 1) in the sheets were created using standard arch punch sets. A total number of holes in a sheet are shown by *m* oriented in a regular pattern. For each group, eight specimens were tested. The number of specimens per group was determined based on a preliminary study detailed in the supplementary document.

We used an experimental setup similar to the one previously described in ref.^4^ for the crumpling of Mylar sheets to achieve an isotropic crumpling condition. The setup consists of a net of fishing wires uniformly distributed around the crumpled sheet. The wires go through a hole and are attached to a bucket that will be filled with different weights. Eight varying levels of weights were gradually added to the bucket, which resulted in stepwise crumpling of the specimens. The samples were allowed to relax for 3–5 min after adding each weight in order to reach the equilibrium. For every step, images were taken from the crumpled specimens using a digital camera. The images were taken in two perpendicular planes. A circle was fitted to the crumpled specimens to measure their size and, thus, their deformation. The average of the diameters measured in two planes was considered as the size of the sample (Fig. 1). To validate the robustness of the experiments, Intra- and inter-observer analysis was also performed (see the supplementary document).

To study the role of hole edges on crumpling process, we used micro-computed tomography (μCT), where two-hole area fraction, i.e. 20% and 40%, were considered and three specimens were tested and scanned for each area fraction. The outer boundaries of the hole in the porous sheets were highlighted by spraying a zinc spray (Motip^®^). Zinc has a different attenuation coefficient as compared to Mylar, meaning that the pore edges could be distinguished with image analysis and the network of their positions could be tracked during the crumpling process. The sprayed zinc ring was created by offsetting the hole edges by 1 mm. The sprayed zinc remained adherent to the sheet as the crumpling process proceeded. A μCT scanner (Quantum FX, Perkin Elmer, USA) was used with a tube current and voltage of 180 μA and 90 kV, respectively. The acquired images had a spatial resolution of 40 μm. The total imaging time was about 2 minutes. The specimens were crumpled to different degrees (various levels of forces) using the same setup as described above. Their crumpling state was then frozen by wrapping a tape sticker around them. The specimens were then scanned in the μCT scanner. After scanning was completed, the sticker tape was removed from the sample, and the crumpling process was continued. Specimens were tested at five different loads. The orientation of the specimens did not change during scanning and loading.

**Figure 2:**
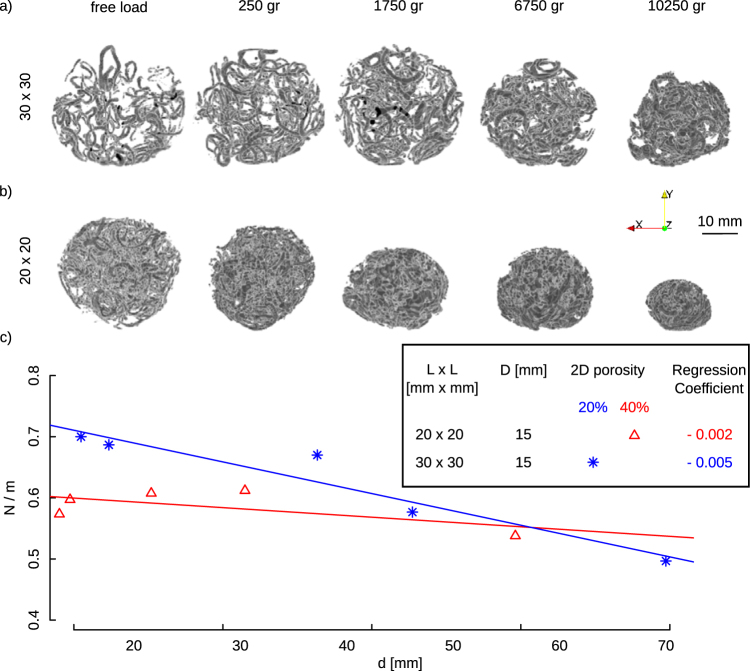
μCT imaging of porous sheets with two levels of porosity of 20% (**a**) and 40% (**b**). The pores in the sheets were highlighted by a conductive spray. The resolution of the μCT image is 40 μm. (**c**) Average of the number of folds from a skeletonize image calculated from three middle planes in the μCT image. The number of folding lines seem to be constant during the compaction.

Image analysis was performed with ImageJ^14^. Using a Gaussian filter (kernel size = 1.5), noises were removed from the image. The Otsu local thresholding method^15^ was used for segmentation of images, which resulted in a binary image with a voxel value of 1 for the conductive ring and 0 for the air and plastic sheets (Fig. 2a,b). Three-dimensional binary images were skeletonized using the relevant plugin in ImageJ^16^. The number of folding lines were measured by averaging the number of particles in the mid-planes of the skeletonized images (Fig. 2c).

The distribution of the ridges and crease lines in both holey and non-holey crumpled sheets was measured by scanning and image analyzing the unfolded crumpled specimens. For that purpose, we scanned five non-holey specimens as well as specimens with 20% hole area fraction using a laser scanner (Ricoh Afico mp c2050) with a resolution of 300 dots per inch (dpi). The ridges and crease lines were identified in the scanned images using image processing algorithms available in ImageJ. The region of interest (ROI) was a square (240.7 mm × 240.7 mm) at the center of the unfolded sheets. A Gaussian filter with a kernel size of 1.5 was applied to minimize the noise present in the scanned images. Images were then converted to an 8-bit format, segmented using an Otsu thresholding algorithm, and skeletonized. We assumed that the length of the pixels in the skeletonized image corresponds to the hinge lines in the crumpled structure.

## Results and Discussion

The force-size curves measured for the specimens from different groups were generally similar in trend and seemed to be merely shifted (Fig. 1a). Once scaled with the ratio of the hole area to the unit cell area, i.e. *A*_0_ */A*_1_, the shift observed in the absolute force-deformation curves disappeared to a large extent and the majority of the data points collapsed into one single trend-line (Fig. 1b). We, therefore, concluded that reduction in the area fraction is the most important factor controlling the crumpling behavior of the holey Mylar sheet. A linear regression model in the logarithmic scale was fitted to the force-deformation data to obtain the power, *n*, of the power law $(F\propto {d}^{n})$ best describing the compaction behavior of the specimens (Table 1). Statistical analysis was performed with R^17^.

**Table 1:** Summary of geometrical parameters for porous, and non-porous Mylar sheets.

Group	Size of the Mylar sheet [mm × mm]	Unit cell size (*L* × *L*) [mm × mm]	*D* [mm]	*A*_0_ [mm^2^]	*A*_1_ [mm^2^]	Initial porosity [%]	Number of pores, *m*	*n*
No por	300 × 300	300 × 300	0	100	100	0	0	6.59
No por-20	268 × 268	268 × 268	0	100	100	20	0	6.39
No por-40	232 × 232	232 × 232	0	100	100	40	0	5.60
UC1010-10	300 × 300	10 × 10	4	100	87.43	10	900	5.22
UC1010-20	300 × 300	10 × 10	5	100	80.37	20	900	6.13
UC1010-40	300 × 300	10 × 10	7	100	61.52	40	900	6.69
UC2020-10	300 × 300	20 × 20	7	400	361.52	10	225	6.09
UC2020-20	300 × 300	20 × 20	10	400	321.46	20	225	6.97
UC2020-40	300 × 300	20 × 20	15	400	223.29	40	225	7.31
UC3030-10	300 × 300	30 × 30	10	900	821.46	10	100	6.26
UC3030-20	300 × 300	30 × 30	15	900	723.29	20	100	6.60
UC3030-40	300 × 300	30 × 30	20	900	585.84	40	100	6.88

These parameters are schematically presented in Fig. 1. Crumpling exponent, *n*, is calculated by fitting a power-law model in $F\propto {d}^{n}$. The absolute value for *n* is presented in this table.

Crumpling a piece of paper into a ball and crushing the structure increases the density by reducing the radius of the ball. It is well known that the average number of layers increases in a power-law manner with the size which results in a power-law dependence of the force required for the compaction of the structure with its size^4^. It has been shown that a simple folding model could capture the important features of disordered crumpling, including the above-mentioned power-law dependence. For a sheet, folded n times repeatedly, the number of the layers and the effective bending rigidity of the structure grows exponentially with the number of folding events n. Translating the number of folding events into the final size of the structure explains the power-law dependence of the number of layers and compaction force on the size^8^.

We therefore expected to have a power-law dependence between the force and size in our system. By plotting the force and the size in a log-log scale we observed the expected behavior. However, for the first two data points in each series, corresponding to small compaction forces at the beginning of the process, we observed deviations from the expected trend. The exponents are obtained by fitting power law to the experimental data. The fitted curves are represented by straight lines in log-log scales where the slope of the lines give the exponents.

At the beginning of the compaction process, when the compaction force is small, the crumpled structure is not spatially isotropic and the average number of layers is only 2 or 3 layers. Therefore, the force distribution around the structure is not uniform. This causes smaller average size and deviation from the power law expected for the force-size behavior, as shown in Fig. 1. By increasing the compaction force, the average number of layers increases and we achieve more isotropic structures which consequently satisfy the power-law trend, as it is expected.

**Figure 3:**
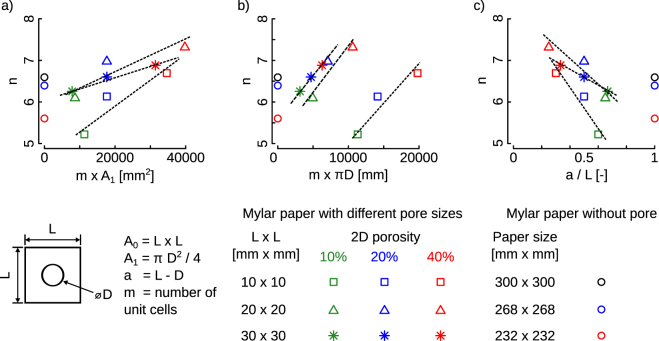
Comparison of the crumpling exponent, *n*, with respect to (**a**) total area fraction reduction, (**b**) total perimeters of pores in the sheets, (**c**) ligament slenderness ratio, *a/L*. Absolute value for *n* is presented here. *Fitted lines are only drawn to clarify the general trends and as guides for the eye*.

The crumpling exponents, *n*, laid between the relatively limited range of 5.22 to 7.3 (Table 1). The crumpling exponents calculated for the normal Mylar sheets in this study are within the range of those reported in ref.^4^. These observations further support the conclusion that the area fraction merely scales the crumpling behavior of holey sheets to a large extent similar to that of the normal sheet. There are, however, some secondary effects that arise due to the presence of the hole edges. For example, there is a clear trend in the crumpling exponents where for a unit cell size, the crumpling exponent increases with both hole area fraction (Fig. 3a) and the perimeter of the hole edges (Fig. 3b). The increased crumpling exponents indicate that the force required for crumpling increases more rapidly for the sheets with more holes. This could be attributed to the effects of interlocking of hole edges and increase the frictional contacts that require more force to overcome as compared to the crumpling of normal sheets. We also defined ligament slenderness ratio as *a/L* similar to slenderness ratio in the buckling of columns. Ligament slenderness ratio equals to 1 shows a sheet without a hole. This parameter shows an inverse trend with respect to the crumpling exponent (Fig. 3c).

The number of folds, *N*, related to the hole edges increased with the compaction level (i.e. the size of crumpled object *d*) (Fig. 2). This is expected and is consistent with what has been found before for normal sheets where the total number of layers in the crumpled structure has been found to increase the compaction level^4,6,8,18^. Once scaled with respect to the number of holes in the sheet, *m*, the trend lines cross each other for large *d* values (Fig. 3b), indicating similar starting points. The rate of increase in the number of edge-related folds is, however, around 2.5 times slower in sheets with higher hole area fraction (Fig. 2b). This indicates that fewer hole edges fold in sheets with higher hole area fraction, while the crumpling process proceeds (Fig. 2b). Given that increased hole area fraction slows down the process of hole-edge folding, alternative paths that are energetically less favorable have to be found for the crumpling process to proceed. Those less energetically favorable pathways could explain the increase in the crumpling exponent with hole area fraction (Fig. 3a).

**Figure 4:**
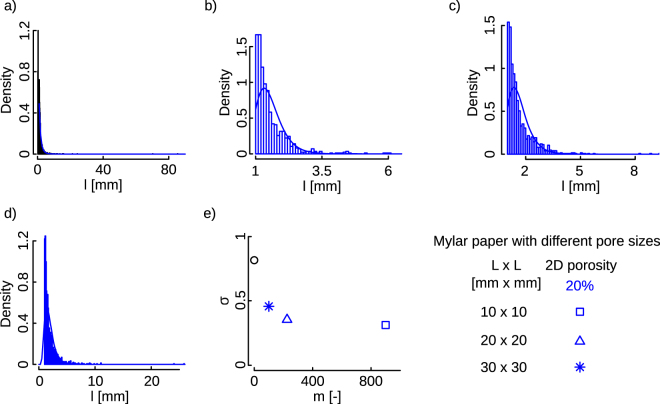
Histogram of distribution of ridges for (**a**) non-porous, (**b**) 10 mm × 10 mm, (**c**) 20 mm × 20 mm, (**d**) 30 mm × 30 mm unit cells. A similar level of areal fraction reduction equal to 20% was chosen for the holey sheets. Histograms show the pooled data for each specimen which contain five samples. The solid lines overlaid on the histogram show the log-normal distribution fitting. (**e**) The standard deviation calculated from the log-normal fit for different porous and non-porous Mylar sheets.

Analysis of the crease line and ridge patterns indicates that the length of the lines is generally smaller in crumpled holey sheets as one expects (Fig. 4a–d). The hierarchical folding mechanism which occurs during the crumpling process supposed to show log-normal distribution. However, it has been previously shown that at high compaction rate the hierarchical nature of crumpling is influenced by self-avoid interactions^19^. To obtain the distribution of the ridges in different specimens a log-normal distribution was fitted to the histogram of the hinge length using the fitdistplus package in R^20^. The log-normal distribution was computed using the pooled data for each group as follows:

1 $$f(l)=\frac{1}{l\sigma \sqrt{2\pi }}{e}^{-\frac{{(lnl-\mu )}^{2}}{2{\sigma }^{2}}}$$

where *l* is the hinge lines, and *μ* and *σ* are the mean and standard deviation, respectively. The standard deviation calculated for the log-normal distributions, *σ*, decreases as the number of holes increases (keeping the initial hole area fraction constant) (Fig. 4e). Both above-mentioned observations suggest that hole edges act as boundaries limiting and guiding the propagation of crease lines and ridges. Several previous studies have investigated the patterns of crease lines and ridges in normal crumpled sheets^5,7,18,21^. By extrapolating the results of the log-normal distribution reported in ref.^7^ to obtain the standard deviation for sheets with a thickness of 30 μm, one could obtain a standard deviation, *σ*, of 1.14. This value is calculated for a different material (i.e. paper) and could be compared with the value we obtained for our normal Mylar sheets (i.e. 0.82) (Fig. 4e).

## Conclusion

In summary, we found area fraction of the hole to be the dominant factor influencing the crumpling behavior of holey thin sheets. The force-deformation curves were largely similar when scaled with respect to the hole area fraction and the crumpling exponents laid in a relatively small range. Pore edges were, however, found to affect the crumpling behavior to some extent. In particular, the crumpling exponents increased with increasing the area fraction and total hole perimeter. Moreover, increased hole area fraction limited the formation of the hole edge folds. Finally, hole edges seemed to act as boundaries limiting and guiding the propagation of crease lines and ridges within the sheet.

## Electronic supplementary material

Supplementary document
