# Supplementary material for: Crumpling-based soft metamaterials: the effects of sheet pore size and porosity
Source: Sci Rep. 2017 Oct 12;7:13028. doi: 10.1038/s41598-017-12821-6 (PMC5638806; doi:10.1038/s41598-017-12821-6)
Supplement: Supplementary file 1 — Supplementary document [file 41598_2017_12821_MOESM1_ESM.doc]

*Supplementary document to*

Crumpling-based soft metamaterials: the effects of sheet pore size and porosity

M. J. Mirzaali^a,b^,[[1]](#footnote-2), M. Habibi^c^, S. Janbaz^b^, L. Vergani^a^, A.A. Zadpoor^b^

*^a^Department of Mechanical Engineering, Politecnico di Milano, Via La Masa 1, 20156 Milano, Italy*

*^b^Department of Biomechanical Engineering, Faculty of Mechanical, Maritime, and Materials Engineering, Delft University of Technology (TU Delft), Mekelweg 2, 2628 CD, Delft, The Netherlands*

*^c^Physics and Physical Chemistry of Food, Department of Agrotechnology and Food Sciences, Wageningen University, The Netherlands*

Based on the random nature of the experiments, a variation in the results was observed. The mean ± standard deviation of the compaction level under different level of applied forces for all the specimens are presented in Table 1s.

**Table 1s: The mean ± standard deviation for the level of compaction at different levels of loading for all specimens.**

| Force [N] |  |  | | *d* [mm] | | | | | | | | |
| --- | --- | --- | --- | --- | --- | --- | --- | --- | --- | --- | --- | --- |
|  | No por | No por-20 | No por-40 | UC1010-10 | UC1010-20 | UC1010-40 | UC2020-10 | UC2020-20 | UC2020-40 | UC3030-10 | UC3030-20 | UC3030-40 |
| 0.50 | 50.9±5.0 | 51.1±8.1 | 43.1±9.9 | 37.8±3.4 | 41.6±3.7 | 33.4±1.6 | 40.7±5.1 | 36.2±3.7 | 29.7±2.0 | 50.1±6.6 | 46.9±4.0 | 38.6±4.5 |
| 1.50 | 49.7±4.2 | 49.6±7.0 | 43.0±8.9 | 37.5±3.4 | 41.0±3.3 | 32.7±0.8 | 40.4±4.8 | 35.3±4.3 | 29.5±1.9 | 47.9± 5.6 | 45.2 ±2.8 | 38.5± 3.2 |
| 2.50 | 49.3±2.9 | 48.5±6.1 | 42.5±8.6 | 37.2±3.3 | 37.6±4.7 | 31.1±2.7 | 39.4±3.4 | 35.4±3.8 | 27.6±2.3 | 44.4±3.1 | 40.2±3.5 | 35.8±3.3 |
| 7.50 | 45.5±4.3 | 43.8±4.4 | 40.2±6.2 | 35.9±3.4 | 34.8±4.8 | 29.0±2.7 | 37.4±2.5 | 33.8±3.3 | 26.4±3.0 | 40.7±3.8 | 36.6±3.1 | 32.3±3.1 |
| 17.50 | 38.1±2.9 | 38.9±2.1 | 35.4±2.7 | 32.8±1.3 | 32.3±3.5 | 26.5±2.0 | 35.1±2.2 | 31.0±3.3 | 23.9±1.8 | 38.0±3.7 | 33.4±2.4 | 30.0±3.1 |
| 37.50 | 34.8±2.8 | 33.8±2.0 | 31.4±2.6 | 29.9±1.8 | 27.9±3.5 | 23.1±2.2 | 31.3±2.1 | 28.7±2.9 | 21.7± 1.8 | 33.1± 2.5 | 28.8±3.2 | 26.8±1.9 |
| 67.50 | 30.5±3.4 | 30.1±2.3 | 26.8±2.1 | 27.5±1.9 | 25.3±2.9 | 20.3±1.8 | 28.1±2.0 | 25.3±2.7 | 19.1±1.2 | 28.8±1.2 | 26.3±2.5 | 23.5±1.4 |
| 102.50 | 28.0±1.5 | 26.9±1.8 | 24.3±2.4 | 25.6±1.4 | 23.1±1.8 | 18.0±1.9 | 25.5±1.6 | 23.3±2.4 | 17.1±1.5 | 24.5±1.8 | 24.1±1.8 | 21.0±2.2 |

To find out the maximum number of samples in order to reach a convergence in the crumpling exponent, we created four more specimens and added them to the current eight specimens that were tested in the group UC3030-40. We calculated the crumpling exponent by analyzing different permutations of these twelve specimens. 1000 simulations were performed to take into account all possible combinations. The difference between the mean value for the crumpling exponent calculated from fitting a power-law on twelve specimens and eight specimens were less than 2%. Similar repetition analyses have been done for the samples in other groups. This analysis justified the maximum number of specimens (eight sample in this study) required to reach a convergence in the crumpling exponent.

All tests have been done by one observer. As we are not following a standard test set up, and the test procedure seems to be subjective to the observer and initial condition, we performed inter- and intra-observer reliability analysis. For the inter-observer analysis, same experimenter repeated three tests for the samples in group UC1010-40 which this group was randomly selected. The mean ± standard deviation for the crumpling exponent showed 0.45 % difference. As for the intra-observer reliability analysis, two more observers were asked to repeat and follow same test protocols this time for the samples in the group UC3030-20. Each observer performed three more tests. The crumpling exponent obtained from the tests of observer 2 and observer 3 was in less than 10% difference that what obtained by the observer 1.

**
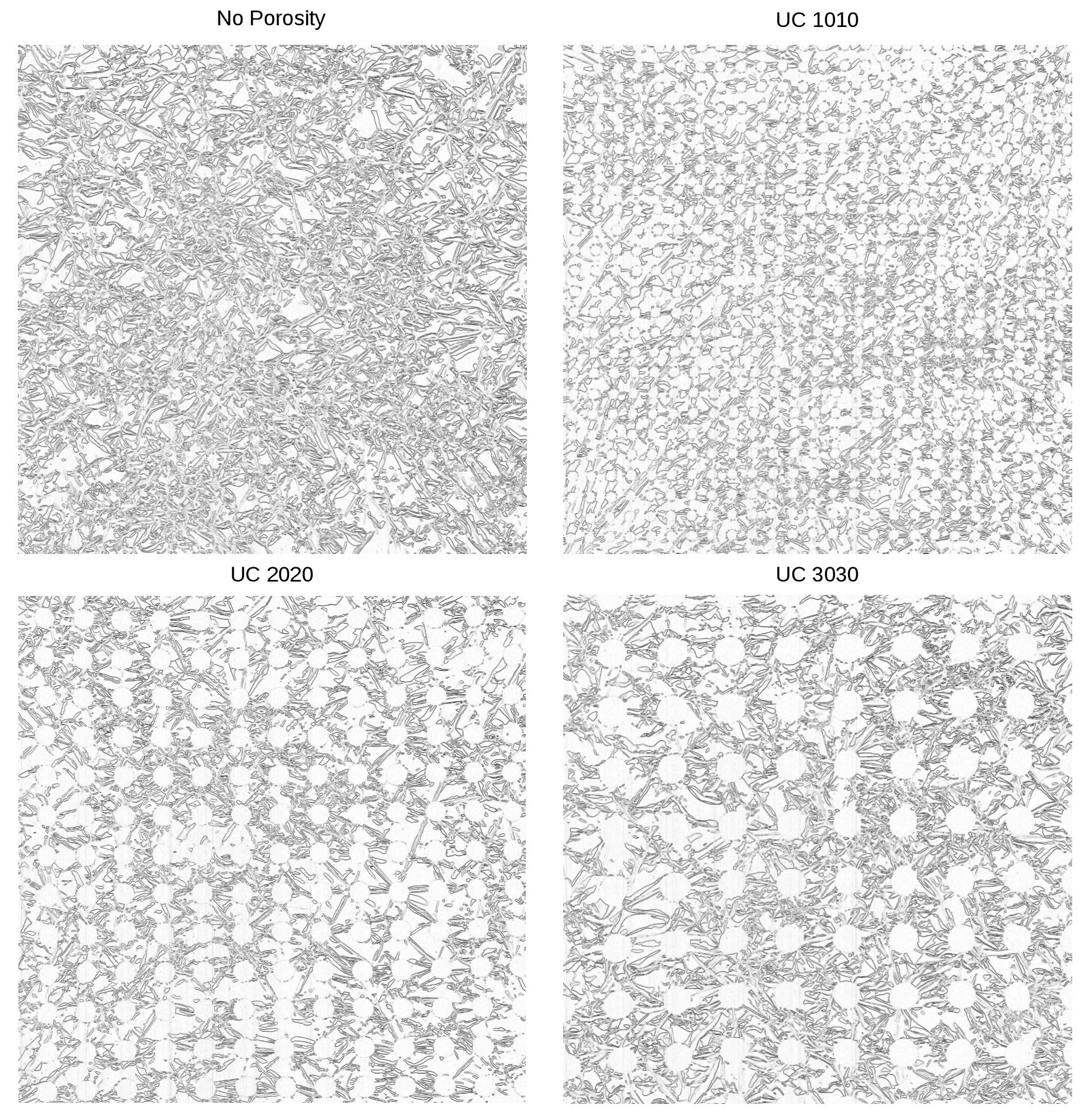
**

Figure 1s. 2D scans of Mylar sheets with different pore size.

1. ?Corresponding author. Tel.: +390223998203; fax: +392 2399 8263.

   *E-mail address:* [mirzaalimazandarani.mohammad@polimi.it](mailto:mirzaalimazandarani.mohammad@polimi.it); mirzaali.mohammad@gmail.com [↑](#footnote-ref-2)
